# Polio’s Precarious Future A Review of *Polio: The Odyssey of Eradication* and an Interview with Dr. T. Jacob John

**DOI:** 10.4269/ajtmh.19-0046

**Published:** 2019-03

**Authors:** Claire Panosian Dunavan

**Affiliations:** Division of Infectious Diseases; University of California; Los Angeles, California; E-mail: cpanosian@mednet.ucla.edu

Dateline 1957. At Brentwood Elementary School, I gravely receive Jonas Salk’s miracle shots, today known as inactivated polio vaccine (IPV). Five years later, at Paul Revere Junior High, I crunch sugar cubes dotted with pink along with other Angelenos who, between 1962 and 1963, collectively ingest 7.5 million doses of Albert Sabin’s oral polio vaccine (OPV). By now, the upside is clearer. With my own eyes, I have seen children wearing awkward leg braces and read about iron lungs. Soon after my birth, the latter, boiler-like contraptions filled entire wards of Los Angeles County-USC hospital during my city’s last, great polio epidemic.

At the same time, the science barely penetrated my brain. It was not until the early 1970s that I fully grasped that OPV—a product containing live attenuated poliovirus types 1, 2, and 3—proliferated in the gut. As a result, our medical school professors proclaimed, OPV not only provided long-term humoral immunity against the paralytic disease but quickly induced short-term intestinal immunity to polio! And all for a fraction of IPV’s cost! Last but not least, in unhygienic settings beyond our borders, the fecal–oral spread of OPV serendipitously protected even more children and adults who would otherwise fail to receive the vaccine.

So, it was really no surprise when, in 1988, public health leaders energized by the previous decade’s success against smallpox embraced OPV as the tool by which the world would evict yet another devastating scourge. As cases plummeted, for a while it even seemed possible to eradicate polio by the year 2000. Then came the creeping awareness that OPV was neither entirely benign nor as reliably effective as hoped in children in the tropics. Bottom line: The target date for polio’s death knell continued to slide.

Today, sadly, eradication is still not assured. On November 30, 2018, a WHO committee met for the 19th time in four years only to deem polio, yet again, a public health emergency of international concern—a dubious honor previously accorded to H1N1 flu (2009), Ebola (2014), and Zika (2016).

The worst-case scenario in the near term? That, in 2019 and beyond, the global south will face double trouble if wild poliovirus type 1 infections in Pakistan and Afghanistan continue to stay flat or rise while circulating vaccine-derived polioviruses (VDPVs) also ratchet up and spread. In 2017 and 2018, paralytic cases caused by mutants of OPV type 2—a strain included in OPVs until 2016, when type 2 was deleted from the standard OPV mix used in 155 countries—were detected in the Democratic Republic of Congo, Niger, Nigeria, Somalia, and Syria. This confirms that neurovirulent OPV variants are still circulating, largely silent and undetected, in multiple sites worldwide where they can potentially afflict under- or unvaccinated people.

The time has come to reexamine the modern polio era. In *Polio: The Odyssey of Eradication*, recently published by Hurst and distributed by Oxford University Press, science journalist Thomas Abraham does just that, first narrating polio’s historical and virologic backstory, and then interrogating its still-elusive eradication. Along the way, the author weaves actors from past and present researchers, to global health leaders fueled by ego and dreams, to passionate, committed Rotarians and local stakeholders. All the while, Abraham pledges first and foremost to represent “the needs and aspirations of those in developing countries” and explicate “the messy reality that lies underneath an ambitious global health campaign, a reality the public rarely sees.” By quoting a rural Nigerian farmer from Kano Province who poignantly asks: “Why do you keep coming again and again to give polio vaccine? Why this polio, polio?” the author echoes the question of millions who cannot quite fathom why a seemingly rare disease commands such an inordinate investment of time, effort, and money, while children in low-income countries still die—day-in, day-out—of measles, malaria, and other deadly, ubiquitous blights.

In the closing chapters of *Polio: The Odyssey of Eradication*, Abraham moves to recent geopolitical events: disinformation around polio vaccine, violence unleashed on polio vaccinators, and the bitter fruit of using vaccine-related subterfuge to locate Osama bin Laden in Abbottabad, Pakistan. But by this point, many readers will have concluded that the stage was already set for a lingering (and possibly unwinnable) war against polio thanks to assorted missteps and the world’s tortoise-like response to OPV’s sporadic mutation to a neurovirulent pathogen. All of these factors have handicapped polio’s final purge.

Offsetting the gloom is polio’s stunning 99 percent decline over three decades—a true cause for celebration. In Abraham’s excellent marriage of narrative, reporting, and commentary, paradoxes abound. As a result, especially for tropical medicine and global health “newbies” seeking hard, nuanced truths, this book is highly recommended.

After finishing *Polio: The Odyssey of Eradication,* I wrote to a physician-researcher featured in its pages. Some have called Dr. T. Jacob John, an Indian pediatrician, medical virologist, and vaccine expert, “near-prophetic” for anticipating roadblocks in the polio endgame long before others either recognized or addressed them. As a record of intellectual independence, his long list of peer-reviewed publications speaks for itself.

Early in our email exchange, however, one question I posed to John involved the dearth of women in the highest polio policy circles, a fact that may seem off-topic to some—to others, both significant and unfortunate. In his answer, John affirmed the gender imbalance and expressed basic philosophic principles. He also shared long-standing dismay over certain features of the Expanded Programme on Immunization [EPI] writ large and inherent flaws in the “OPV-only strategy” that, until recently, prevailed in the Global Polio Eradication Initiative [GPEI].

## INTERVIEW WITH T. JACOB JOHN

Let me start by responding to your question about women in GPEI leadership. I look at it this way. I grew up learning do no harm to any living creature—not even small animals—except for pests that are harmful. I was also taught never to exploit the “vulnerable,” namely, the ones who trust and depend on you. Then, in medical school, we learned *primum non nocere.*

So when I looked at IPV and OPV, all I had to ask was this: Were they equal in efficacy but unequal in safety? If OPV was more efficacious but a little unsafe, then one could justify its promotion. The answer I found for us in India was that OPV was both inefficient and universally unsafe in spite of its being branded as “one of the safest…,” while IPV was both completely safe and exquisitely efficient. The OPV-only position of GPEI, and earlier of EPI, went against my conscience and convictions. My guess is that women might have had similar lines of thinking that were more compassionate, more humane, and more ethical. If so, we would have eradicated polio quicker and cheaper.

As for EPI, from its earliest days, I argued that any system should have inputs, processes, and output, plus evaluations at all levels. Coverage as the only evaluation was not acceptable to me. Disease reduction and “demand creation” were two further outputs (I noticed that in Syria there was demand for OPV from parents as soon as the VDPV outbreak was detected). For the former, public health surveillance was necessary. So I designed one for Kottayam district in the state of Kerala and also tried to promote that concept in EPI.^1–3^ To date, unfortunately, there has been no progress. EPI still remains a vaccine delivery platform.

**Now let’s stand back. Please summarize the “intertwined” history of the Expanded Programme on Immunization and the Global Polio Eradication Initiative. In addition, based on experience in India, what is the best way to “reach all children” with polio and/or EPI vaccines?**

Originally, polio eradication was to be achieved through the global EPI [note: first established in 1974, the WHO’s EPI program was originally meant to dramatically boost the global coverage of children with BCG, polio, tetanus, pertussis, diphtheria, and measles vaccines], an approach that would have forced all countries to find and correct flaws in the system. And, in fact, for several years polio eradication was managed by EPI, but later on the plan shifted to an independent Global Polio Eradication Initiative (GPEI) based at WHO. GPEI realized that OPV given under EPI plus an annual campaign or two would not be sufficient to eradicate polio in many tropical countries. More polio campaigns were necessary, far more than EPI would have been able to conduct. Moreover, clinical and virological surveillance was essential, but EPI had no capacity to build it in. Hence GPEI had to be de-linked from EPI.

On the other hand, if IPV had been “allowed” for polio eradication, then EPI still could have played a key role and, quite likely, one or two OPV pulse campaigns per year would have been sufficient to interrupt wild poliovirus transmission. The reduction in polio cases would have been strictly proportional to IPV coverage. When necessary, OPV campaigns could be added. For such a strategy, both IPV and OPV should have been used in a relay sequence. IPV first and then OPV would have prevented vaccine-associated polio due to OPV and also retarded the evolution of virulent VDPVs.

But that was not to be; GPEI remained “allergic” to the idea of using IPV until 2012. The overall result was that EPI was not strengthened when it should have been. Now that IPV is required for the conclusion and completion of eradication, the job has fallen to EPI. The worry is that we may not get high enough coverage for securing eradication in many countries, especially, as of now, in India.

EPI is in urgent need of strengthening. Not only for polio eradication but also to achieve control of all EPI vaccine-preventable diseases, EPI has to be reengineered as a disease control program. Immunization should become an integral part of primary health care, for which purpose universal primary (and secondary) health care must be provided. Sri Lanka is a model.

Without giving them what they need and deserve, governments cannot expect people to simply accept what is provided, and that includes immunization. Immunization costs families time and travel, but what is the benefit? The benefit is both deferred and invisible—hence the crucial need for effective health education.

**In your view, is there a better way to coordinate the global switch to IPV as opposed to country-by-country decision-making?**

I would not use the term “switch” as it was applied to the 2016 switch from trivalent OPV to bivalent OPV, which suggests globally synchronous action. IPV is already accepted in the endgame strategy, so all countries are now IPV licensed. The latest requirement is for “at least one dose.” Although GPEI has not explicitly stated so, the polio-eradicated world will have to include IPV in all national EPI schedules with a “minimum of two” well-timed doses so that immunity is both near 100% and long lasting. If a country has such a schedule and reaches very high coverage—say 90% and more—there is no reason for that country to continue to give OPV. In my mind, a schedule that results in near 100% antibody response and coverage exceeding 90% are the only two requirements for any country to unilaterally withdraw all OPV, which currently means bivalent OPV. New Zealand, Japan, and Malaysia have already done just that. However, each country should also participate in high-quality clinical surveillance for acute flaccid paralysis (AFP) as well as virological surveillance, both for the safety of the country and for the rest of the world.

What I personally advocated for and was instrumental in establishing in India in the late 1980s was an IPV manufacturing company. But the government closed it down within a couple of years, misguided by global advice that IPV would never be used or required for polio eradication. Such obstinacy was indeed unfortunate. Ever since then, I declared that, without IPV, global polio eradication would not be possible.

Today the main problem is an inadequate supply of IPV; the second problem is its slowly increasing price. Any country that can afford and procure sufficient vaccine must move in this direction. There is no risk involved in introducing IPV. When coverage is high, there is also no risk in removing OPV. The larger the number of countries that replace OPV with IPV, the earlier we can complete and conclude global polio eradication.

**Turning to contemporary challenges, what do you make of the recent surge in cases of acute flaccid myelitis (AFM) in the United States?**

We must always expect the unexpected. Acute flaccid paralysis due to myelitis is an example. Several enteroviruses cause CNS diseases; viral meningitis is the most common, whereas myelitis with paralysis is rare. Enterovirus 70, which emerged in 1968, causes hemorrhagic conjunctivitis and polio-like paralysis. Enterovirus 71 can cause encephalitis and acute flaccid paralysis. Enterovirus D68 has appeared in recent years and been found to cause a few cases of polio-like paralysis. In terms of causality, it’s my understanding that the cause of the current upsurge of AFM in many states in the United States remains unknown. It’s a good thing there’s no polio to confuse it with. However, the absence of polio does not prove the niche theory—that some disease will eventually occupy the niche vacated by polio. No doubt the cause will be discovered in the United States, but just imagine if this problem occurred in a country or countries with no surveillance or competence to detect and diagnose.

The lesson for all of us is that new diseases will continue to appear from time to time. Universal health care, public health surveillance, and local access to diagnostic lab services are three pillars that must be established in all countries.

In 1974, EPI was created in a virtual vacuum. So why, today, do we not raise the bar and establish universal health care plus public health as the reengineered EPI? EPI was meant to fill the gap in primary health care and public health. In 2018, the same gaps remain. Unless these two gaps are deliberately and by design covered, EPI will simply remain a silo of vaccine delivery.

**Are you beginning to see a significant “anti-vaxxer” movement in India? What’s the best way to combat this trend in low- and middle-income countries as well as industrialized nations?**

There have always been self-appointed opponents of anything that is good. In the vaccine arena, there are two types, starting with a few people who genuinely believe that vaccines are unwanted, harmful, and serve only to profit manufacturers. Some of this group have experienced an unfortunate event, like a child dying soon after immunization, and erroneously attribute that event to the vaccine. They seem to be in the minority.

The other type are irrational. Their beliefs do not stem from the cognitive realm and are not easily overcome with factual information. They are vocal and argumentative and try to dissuade parents from immunizing their children. They work on the fear principle. We do have our share of such individuals in India. What we have done through an organization called the Child Health Foundation is to provide high-quality vaccine education for government immunization officers and pediatric academics and practitioners so that, in their own contexts, they become true experts to combat disinformation and misinformation.

Perhaps school curricula should also offer histories of diseases that were controlled with vaccines so that children learn to respect vaccines. This teaching would be based on factual, recorded information. If you go beyond that and promote immunization, then I suspect the anti-vaccine zealots would cry foul.

All of us have to be vigilant and prompt. Social media make it easy for misinformation to spread. Not long ago, our local high court decreed that if you forward a Whatsapp message, it will be assumed you knowingly endorse its content. This should discourage people from glibly forwarding messages and misinformation. In today’s world, vigilance and quick remedial action are required by all who know the benefits of immunization.

***Addendum: The following highlights key events in Dr. John’s professional career.***

After receiving my MBBS and DCH (Diploma in Child Health) in India, in 1964, I went to the United Kingdom and passed the MRCP exam, leading to membership in the Royal College of Physicians (Edinburgh). From 1964 to 1966, I completed a two-year infectious diseases fellowship at the University of Colorado while, at the same time, earning my M.Sc. degree. Training in India and the United Kingdom gave me excellent clinical skills. Training in the United States prepared me both to “think” and “write” science.

Upon returning to Christian Medical College in Vellore, I originally intended to do pediatric infectious diseases but institutional need and my M.Sc. led me to Microbiology. As a result, in 1967–68 I converted a small research virology unit in Pediatrics into a full-fledged Diagnostic Virology Unit that led to many “firsts,” including detecting the failure of the original 3-dose OPV schedule, detecting HIV infection in India, and diagnosing [post-measles] subacute sclerosing pan-encephalitis, among others.

In 1979, the Indian Council of Medical Research recognized our Virology Unit as a “Centre of Excellence.” In 1986, soon after we detected HIV, I had the unique opportunity to design a national, multipronged response, including annual, repetitive anonymous HIV antibody testing of men with sexually transmitted diseases and antenatal testing of women—the polar extremes of high- and low-prevalence HIV infection. As a result, India has continuous data on the prevalence of HIV infection that dates from 1986 until now, allowing us to gauge India’s success over time in controlling HIV. Our Centre of Excellence was now, in addition, the National HIV/AIDS Reference Centre.

I served as Director of the ICMR Centre of Advanced Research (from 1979) and of the HIV Reference Centre (from 1986) until December 1995, when I retired as head of the Department of Clinical Virology. I was elected President of the Indian Association of Medical Microbiologists in 1994 and President of the Indian Academy of Pediatrics in 1999. I am also a Fellow of the Indian National Science Academy and the Indian Academy of Medical Sciences. Finally, I remain an active Rotarian; in 1984, I was a Founding Member of the Rotary PolioPlus Committee.

At present, my professional activities in public health are reduced but some crucial ones continue, including my three-decade-old advocacy and “activism” for introducing Salk vaccine globally, which partly succeeded in 2015. Now I am working to discontinue Sabin vaccine globally and to replace it with two or three doses of Salk vaccine for every child. Next in line for elimination in India are measles and rubella. I currently chair the Expert Group for the Indian government. Our activities started two years ago; we hope the tempo will pick up in the near future.
